# Activation of M1 muscarinic acetylcholine receptors by proline-rich oligopeptide 7a (<EDGPIPP) from *Bothrops jararaca* snake venom rescues oxidative stress-induced neurotoxicity in PC12 cells

**DOI:** 10.1590/1678-9199-JVATITD-2023-0043

**Published:** 2024-02-09

**Authors:** Carlos Alberto-Silva, Halyne Queiroz Pantaleão, Brenda Rufino da Silva, Julio Cezar Araujo da Silva, Marcela Bermudez Echeverry

**Affiliations:** 1 Natural and Humanities Sciences Center (CCNH), Experimental Morphophysiology Laboratory, Federal University of ABC (UFABC), São Bernardo do Campo, SP, Brazil.; 2 Center for Mathematics, Computation and Cognition (CMCC), Federal University of ABC, São Bernardo do Campo, SP, Brazil.

**Keywords:** Bothrops jararaca, Neuroprotection, Bioactive peptide, Proline-rich oligopeptide

## Abstract

**Background:**

The bioactive peptides derived from snake venoms of the Viperidae family species have been promising as therapeutic candidates for neuroprotection due to their ability to prevent neuronal cell loss, injury, and death. Therefore, this study aimed to evaluate the cytoprotective effects of a synthetic proline-rich oligopeptide 7a (PRO-7a; <EDGPIPP) from *Bothrops jararaca* snake, on oxidative stress-induced toxicity in neuronal PC12 cells and astrocyte-like C6 cells.

**Methods:**

Both cells were pre-treated for four hours with different concentrations of PRO-7a, submitted to H_2_O_2_-induced damage for 20 h, and then the oxidative stress markers were analyzed. Also, two independent neuroprotective mechanisms were investigated: a) L-arginine metabolite generation via argininosuccinate synthetase (AsS) activity regulation to produce agmatine or polyamines with neuroprotective properties; b) M1 mAChR receptor subtype activation pathway to reduce oxidative stress and neuron injury.

**Results:**

PRO-7a was not cytoprotective in C6 cells, but potentiated the H_2_O_2_-induced damage to cell integrity at a concentration lower than 0.38 μM. However, PRO-7a at 1.56 µM, on the other hand, modified H_2_O_2_-induced toxicity in PC12 cells by restoring cell integrity, mitochondrial metabolism, ROS generation, and arginase indirect activity. The α-Methyl-DL-aspartic acid (MDLA) and L-N^Ω^-Nitroarginine methyl ester (L-Name), specific inhibitors of AsS and nitric oxide synthase (NOS), which catalyzes the synthesis of polyamines and NO from L-arginine, did not suppress PRO-7a-mediated cytoprotection against oxidative stress. It suggested that its mechanism is independent of the production of L-arginine metabolites with neuroprotective properties by increased AsS activity. On the other hand, the neuroprotective effect of PRO-7a was blocked in the presence of dicyclomine hydrochloride (DCH), an M1 mAChR antagonist.

**Conclusions:**

For the first time, this work provides evidence that PRO-7a-induced neuroprotection seems to be mediated through M1 mAChR activation in PC12 cells, which reduces oxidative stress independently of AsS activity and L-arginine bioavailability.

## Introduction

Venom-derived proteins and peptides have been utilized as a development basis for new therapeutics targeting various voltage-gated channels, ligand-gated channels, membrane transporters, and enzymes [1, 2]. Snake venom compounds have been investigated as treatments for neurodegenerative disorders [1, 3- 11], and an increasing amount of data suggests that peptides derived from natural materials or their synthetic analogs are possible choices among the many different kinds of substances studied as peptides-promising therapeutic candidates for neuroprotection [12]. Neuroprotective activity of low molecular mass fractions obtained from snake venoms of the Viperidae family species, such as *Bothrops atrox* and *Bothrops jararaca*, has been reported in the literature [11, 13, 14]. Components <10 kDa obtained from *B. jararaca* snake venom demonstrated neuroprotective activity against H_2_O_2_-induced toxicity in cultured hippocampal cells, reducing caspase-3 and caspase-8 expressions [11]. In neuronal-like PC12 cells, this fraction also increased cell viability and metabolism against H_2_O_2_-induced neurotoxicity, reducing oxidative stress markers such as reactive oxygen species (ROS) generation, nitric oxide (NO) production, and arginase indirect activity through urea synthesis [14].

The *B. jararaca* snake venom contains a variety of proline-rich oligopeptides (PROs), also known as bradykinin potentiating peptides (BPPs) [15- 18]. These peptides were the first natural angiotensin I-converting enzyme (ACE) inhibitors [19], which contain 5 to 14 amino acid residues with a pyroglutamic residue (<E) at the N-terminal and a proline (P) residue at the C-terminal [16]. In addition, PROs longer than seven amino acids share similar features, including a high content of proline (P) residues and the tripeptide sequence Ile-Pro-Pro (IPP) at the C-terminal [16]. ACE inhibition and bradykinin potentiation were assumed to be the conventional mechanisms behind the hypotensive effects of numerous PROs [20]. However, new biological activities and targets have been described for PROs, such as argininosuccinate synthetase (AsS) activators [21, 22], increase in L-arginine bioavailability [21, 22], and M1 muscarinic acetylcholine receptor (M1 mAChR) agonists [23, 24].

The AsS and argininosuccinate lyase (AsL) enzymes are rate-limiting components in both the urea- and arginine-citrulline cycles [25]. Enzyme AsS catalyzes argininosuccinate formation through aspartate and citrulline conjugation. Argininosuccinate is cleaved by AsL to produce fumarate and L-arginine [25]. Products of L-arginine metabolism represent a wide range of biologically active intermediates that participate in several metabolic and signaling pathways [26- 29]. L-arginine metabolism products like as agmatine and polyamines (spermine, spermidine, and putrescine) are implicated in neuroprotection pathways [26, 27, 30]. The PRO-10c (<ENWPHPQIPP) enhances the generation of L-arginine by regulating AsS activity and expression [21, 31] and it displays neuroprotective action in neuronal SH-SY5Y cells against H_2_O_2_-induced oxidative damage [10]. It has been hypothesized that PRO-10c enhances L-arginine synthesis by activating AsS, and that agmatine or polyamines generation explains its neuroprotective activity [10]. Neuroprotection mediated by distinct PROs against oxidative stress in SH-SY5Y cells was also demonstrated, but some of the mechanisms underlying neuroprotection are independent of AsS activity and L-arginine bioavailability, such as PRO-7a (<EDGPIPP) [9]. 

Peptide PRO-7a is a weak ACE inhibitor [32], but a potential natural agonist of the M1 mAChR and modulator calcium transients in neurons [24]. The mAChR is highly expressed in the central nervous system (CNS), in the cortex, hippocampus, and striatum, key areas of cognition, memory, motor control, and learning [33]. They form one of the G-protein receptor complexes in the cell membranes of certain neurons and other cells are particularly responsive to the natural compound muscarine, and belong to the class of metabotropic receptors that use G-protein coupled receptors [33]. The mAChR family receptor is composed of five subtypes (M1-M5) [34] and has well-known neuroprotective effects in the brain, which are largely related to M1 mAChR receptor subtype activation [33]. The M1 mAChRs are classically coupled to the G-protein family to trigger the activation of phospholipase C (PLC) and protein kinase C (PKC) which inhibit the glycogen synthase kinase 3β (GSK3β) to decrease Aβ and tau hyperphosphorylation and oxidative stress [33, 35]. The M1 mAChR activation has been shown to ameliorate cognitive impairments and change the onset or progression of Alzheimer's disease dementia [35], and it has also emerged as a critical treatment target for neurodegenerative disease [33, 34]. 

The goal of this work was to investigate if synthetic PRO-7a could protect neurons-like PC12 cells with dopaminergic characteristics and astrocyte-like C6 cells from oxidative stress-induced damage. Furthermore, the involvement of two independent neuroprotective mechanisms was investigated: L-arginine metabolite generation via AsS activity regulation, producing agmatine or polyamines with neuroprotective properties; and M1 mAChR receptor subtype activation via protein kinase C (PKC) pathway.

## Material and methods

### Reagents, chemicals, and synthetic peptides

All reagents and chemicals used in the present study were of analytical reagent grade (purity higher than 95%) and purchased from Calbiochem-Novabiochem Corporation (USA), Gibco BRL (New York, USA), Fluka Chemical Corp. (Buchs, Switzerland) or Sigma-Aldrich Corporation (St. Louis, MO, USA). The α-Methyl-DL-aspartic acid (MDLA), L-N^Ω^-Nitroarginine methyl ester (L-Name), and dicyclomine hydrochloride (DCH) were purchased from Sigma-Aldrich Corporation (St. Louis, MO, USA). The stock solutions of these compounds were prepared in solvent-appropriate amounts according to their technical specifications. The synthetic peptide PRO-7a (<EDGPIPP) was purchased from FastBio (Ribeirão Preto, Brazil). The peptide was analyzed by reversed-phase high-performance liquid chromatography (HPLC; Shimadzu, Kyoto, Japan) and MALDI-TOF mass spectrometry (Amersham Biosciences, Uppsala, Sweden), and purity was higher than 98%. 

### Cell lines

Two types of cell lines were used in the present study: astrocyte-like cell line C6 isolated from the brain of a rat with glioma (ATCC® CCL-107™ from American Type Culture Collection - ATCC, Manassas, VA, USA); and neuronal PC12 cell derived from a transplantable rat pheochromocytoma (ATCC® CRL-1721™ from ATCC, Manassas, VA, USA).

### Culture and maintenance

C6 and PC12 cells were routinely cultured in DMEM medium (Sigma-Aldrich, St. Louis, MO, USA), and supplemented with 5 or 10% fetal bovine serum (FBS) (Gibco, Waltham, USA), respectively. All mediums were also supplemented with 1% (v.v^-1^) of 10000 U.mL^-1^ penicillin, 10 mg.mL^-1^ streptomycin, and 25 µg.mL^-1^ amphotericin B solutions (Sigma-Aldrich, St. Louis, MO, USA). The cultures were kept at 37 ºC in a humidified atmosphere containing 5% CO_2_ and 95% air (Water Jacketed CO_2_ Incubator, Thermo Scientific). Culture medium was replaced every 2-3 days, and at 80% confluence, cells were passaged using trypsin-EDTA solution (0.05% (m.v^-1^) trypsin and 0.02% (m.v^-1^) EDTA).

### Cytotoxicity studies

The C6 and PC12 cells were seeded into 96-well plates (Nest Biotechnology, Rahway, USA), at 5 × 10^3^ cells per well. Cells were treated with 10, 1, and 0.01 µM of PRO-7a in a final volume of 0.10 mL. The plate was incubated at 37 °C for 1, 6, 24, and 48 h. For each concentration and time course studied, there were control and dimethyl sulfoxide (DMSO) groups, which represent untreated cells (only one equal volume of the culture medium) and treated with DMSO (5%; v.v^-1^) diluted in the medium culture, respectively. The cytotoxic effects of PRO-7a were determined by the staining of attached cells with crystal violet dye, according to the literature [36]. After the treatment, the medium was aspirated, and the cells were stained with crystal violet staining solution (0.5 %), washed, and air-dried. Then, methanol (200 μL) was added to each well, and the absorbance was measured at 570 nm using a SpectraMax reader (Molecular Devices, CA, USA). Data were obtained from three independent experiments in triplicate, expressed as the mean ± SD, and represent the percentage of cell viability concerning the control.

### Cytoprotection assay in C6 and PC12 cells against oxidative stress

**Figure 1.**
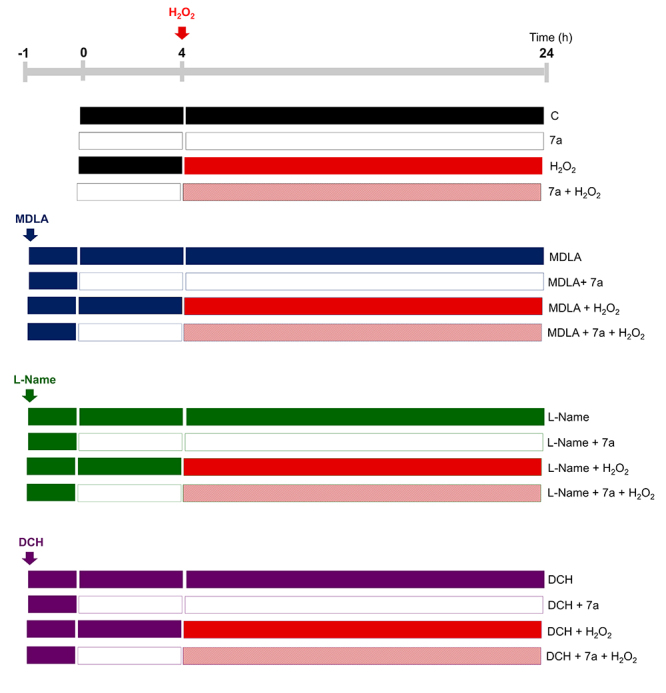
Schematic representation of experimental groups. Cells were pre-treated for four hours at 37 °C with DMEM medium or PRO-7a diluted in DMEM medium. After that, the mediums were replaced by medium containing PRO-7a or/and H_2_O_2_ and incubated for 20 h more. The involvement of L-arginine metabolism and M1 mAChR activation were studied in cytoprotection mechanisms using a specific inhibitor of the AsS (α-Methyl-DL-aspartic acid; MDLA) and a nonselective inhibitor of the NOS (L-N^Ω^-Nitroarginine methyl ester; L-Name), a competitive M1 mAChR antagonist (dicyclomine hydrochloride; DCH). PC12 cells were pre-treated with MDLA (1 mM), L-Name (1 mM), or DCH (10 µM) diluted in DMEM medium for one hour. After, the mediums were replaced by mediums containing or not PRO-7a for four hours at 37 °C, followed by the addition of H_2_O_2_ for 20 h in the presence of compounds.

The cellular stress model used in this work was based on the H_2_O_2_-induced oxidative stress, according to described in the literature [14]. Briefly, C6 or PC12 cells were seeded at 5 x 10^3^ cells per well in a 96-well plate (Nest Biotechnology, Rahway, USA) for 24 h. Then, cells were pre-treated for 4 h at 37 °C with PRO-7a (25 to 0.035 µM), diluted in DMEM medium. After, the mediums were replaced by medium containing the PRO-7a and H_2_O_2_ [0.4 mM in C6 cells; 0.5 mM in PC12 cells ] [14] and incubated for 20 h more (7a + H_2_O_2_ group). Cells untreated (control) or treated with H_2_O_2_ were also incubated under the same conditions (Figure 1). Next, the cytoprotective effects against H_2_O_2_-induced oxidative stress of PRO-7a on C6 and PC12 integrity cells were estimated using crystal violet dye - as described above [36]. If the PRO-7a demonstrated cytoprotective effects in some cell lines, mitochondrial metabolism was also examined using the 3-(4,5-dimethylthiazol-2-yl)-2,5-diphenyltetrazolium bromide (MTT) reduction assay [37]. 

### L-arginine metabolism and M1 mAChR activation in cytoprotection mechanisms

The involvement of L-arginine metabolism was studied using a specific inhibitor of the argininosuccinate synthase (AsS) and also the rate-limiting enzyme for the recycling of L-citrulline to L-arginine [α-Methyl-DL-aspartic acid (MDLA; Sigma-Aldrich, St. Louis, MO, USA)] [10, 31]; and a nonselective inhibitor of nitric oxide synthases (NOS) [L-NΩ-Nitroarginine methyl ester (L-Name; Sigma-Aldrich, St. Louis, MO, USA)] [38]. The M1 mAChR activation was also studied using a competitive M1 muscarinic antagonist, the dicyclomine hydrochloride (DCH) [39]. PC12 cells were seeded in 96-well plates (Nest Biotechnology, Rahway, USA) at 5×10^3^ per well and pre-treated with L-Name (1 mM) or MDLA (1 mM), L-Name (1 mM) or DCH (10 µM) diluted in DMEM medium (100 µL) supplemented with antibiotics and FBS for one hour. After, the mediums were replaced by medium containing PRO-7a (1.56 µM) for 4 h at 37 °C, and then was followed by the addition of H_2_O_2_ (1.5 mM) for 20 h (MDLA + 7a + H_2_O_2_, L-Name + 7a + H_2_O_2_ , DCH + 7a + H_2_O_2_ groups). Cells untreated (control group) or treated with 7a, H_2_O_2_, MDLA, L-Name, DCH, MDLA + 7a, L-Name + 7a, DCH + 7a, MDLA + H_2_O_2_, L-Name + H_2_O_2_ or DCH + H_2_O_2_ were also incubated under the same conditions (Figure 1). Afterward, all groups were analyzed by mitochondrial metabolism, ROS generation, and arginase activity.

### Mitochondrial metabolism assay

Mitochondrial metabolism of PC12 cells in all groups (Figure 1) was analyzed by the MTT. For the MTT assay, cells were treated with 0.5 mg⋅mL^-1^ MTT in the same medium culture for three hours at 37 °C, and the formazan produced was dissolved in DMSO (100 %). The amount of MTT formazan dissolved was determined by measuring absorbance with a microplate reader (Spectramax M3 multi-mode, Molecular Devices, CA, EUA) at 540 nm. Data were expressed as box-and-whisker plots of mitochondrial metabolism percentage concerning the control.

### ROS quantification

ROS generated were assessed using 2’,7’ - dichlorodihydrofluorescein diacetate (H_2_DCF-DA; Sigma-Aldrich, St. Louis, MO, USA) staining, according to the previous procedure [40]. H_2_DCF-DA stock solution was dissolved into anhydrous DMSO before incubation, which was diluted to 1 mM and stored as aliquots in a -20 °C freezer. The stock solution and aliquots were made in the dark. After the treatments, the culture medium of groups (Figure 1) was collected and centrifuged at 9,9391 × *g* for 5 min. Fifty microliters of culture medium were separated and diluted three-fold into PBS solution in a 96-well dark plate (SPL Life Science - Gyeonggi-do, Korea). H_2_DCF-DA was added into each well at a final concentration of 25 µM and incubated for one hour at 37ºC. H_2_DCF-DA fluorescence intensity was measured using a Spectramax device (Molecular Devices, CA, EUA). The excitation filter was set at 480 nm and the emission filter at 530nm. The results of each experiment were reported as mean values from triplicate wells as arbitrary units. 

### Arginase activity

The arginase activity was determined by measuring the metabolite urea, a byproduct of L-arginine degradation from cells, according to the literature [41]. Cells were untreated or treated with 7a, H_2_O_2_, MDLA, L-Name, MDLA + 7a, L-Name + 7a, MDLA + H_2_O_2_, L-Name + H_2_O_2_, MDLA + 7a+ H_2_O_2_ or L-Name + 7a + H_2_O_2_) as described in the procedure above. After, the culture medium was collected, and cells were washed twice with 150 μL of PBS, added 30 μL of lysis buffer (150 mM NaCl, 1% Triton X-100, 50 mM Tris pH 8.0), and incubated for 15 min under agitation at 100 × *g* at room temperature. Subsequently, the medium culture and crude extract protein samples were used to determine the urea concentrations using a Urea analysis kit provided by Roche (Roche/Hitachi cobas c systems; Roche Diagnostics Corporation, Indianapolis, IN) and a microplate reader (Spectramax M3 multi-mode, Molecular Devices, CA, EUA) at 340 nm. A calibration curve was prepared with increasing amounts of urea between 20 and 0.04 mM. 

### Statistical analysis

Data were shown as mean ± SD or box-and-whisker plots of three independent experiments in sextuplicate. Data were analyzed using one-way analysis of variance (ANOVA) for between-group comparisons, followed by Tukey’s post-hoc test for multiple comparisons or Dunnett’s post-hoc test to compare each of several treatments with a single control. Values of p < 0.05 were considered to be statistically significant. The analyses were performed using GraphPad Prism 6.0 software (GraphPad Software, Inc., La Jolla, CA).

## Results

### Toxicological profile of PRO-7a

**Figure 2.**
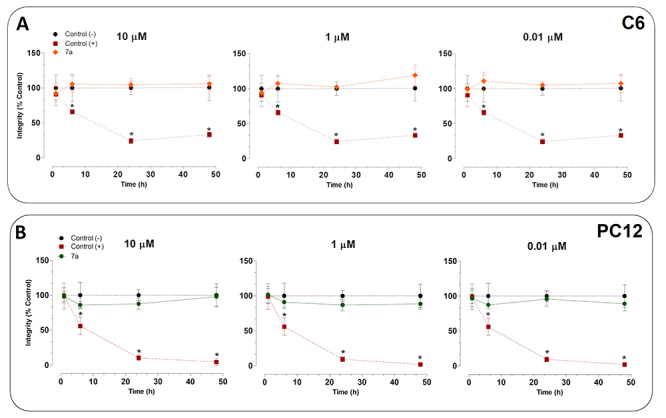
Toxicity of PRO-7a in astrocyte-like, neuronal, and typical fibroblastic. **(A)** C6 and ( **B**) PC12 cell lines treated with PRO-7a at 10, 1, and 0.01 µM for 3, 6, 12, 24, and 48 h. Cells without treatment (negative control) and treated with DMSO 5% (positive control) were included in all experiments. Values are expressed as median ± SD from three independent experiments in triplicate and analyzed by one-way ANOVA followed by Dunnett’s post-test. p < 0.05 vs. control group (*). DMSO: Dimethyl sulfoxide.

Peptide PRO-7a at 10, 1, or 0.01 μM did not impair cellular integrity in two types of cells in conditions tested compared to the control (Figure 2). DMSO at 5% (v⋅v^-1^) reduced cell integrity in PC12 and C6 cell lines in a concentration-dependent manner (Figure 2).

### Cytoprotection in astrocyte-like C6 cells

**Figure 3.**
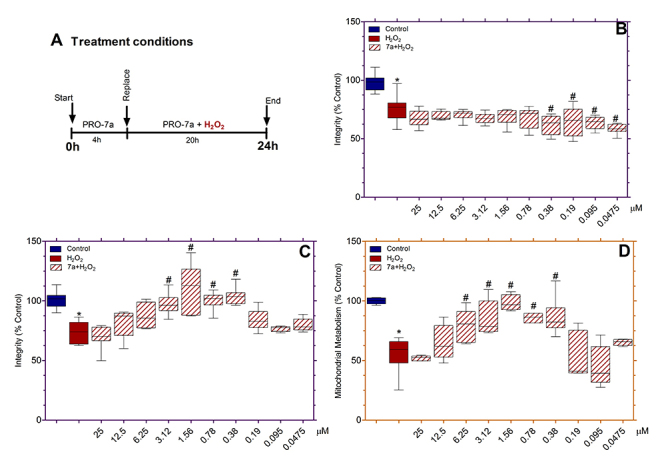
PRO-7a-mediated cytoprotection on oxidative stress-induced changes in astroglial and neuronal cells. **(A)** Cytoprotection model adopted in C6 and PC12 cells. **(B)** PRO-7a effects against oxidative stress-induced neurotoxicity on the integrity of C6 cells. **(C and D)** PRO-7a effects against oxidative stress-induced neurotoxicity on integrity and mitochondrial metabolism of PC12 cells. The cell integrity and mitochondrial metabolism activity were assessed by crystal violet and MTT protocols. Values were presented as box-and-whisker plots from three independent experiments in sextuplicate. Data were analyzed by one-way ANOVA followed by Dunnett’s post-test. p < 0.05 for differences in relation to the control (*); p < 0.05 for differences in relation to H_2_O_2_ (#). MTT: 3-(4,5-dimethylthiazol-2-yl)-2,5-diphenyltetrazolium bromide.

C6 cells were pretreated with PRO-7a at different concentrations ranging from 25 to 0.04 μM for four hours and submitted to H_2_O_2_-induced oxidative stress (0.4 mM) for 20 hours (Figure 3 A). The PRO-7a showed no cytoprotective effects in all concentrations tested but potentiated the H_2_O_2_-induced damage to cell integrity at a concentration lower than 0.38 μM (Figure 3 B). The C6 cells were subjected to oxidative stress with H_2_O_2_ at 0.4 mM to decrease cell integrity to 75.15 ± 2.97% in relation to control. 

### Cytoprotection in neuronal PC12 cells

The cytoprotection model used in PC12 cells was the same as that employed in C6 cells (Figure 3 A). PC12 cells were pretreated with PRO-7a ranging from 25 to 0.04 μM for four hours and then submitted to oxidative stress (0.5 mM) for another 20 h. The PRO-7a at doses ranging from 3.12 to 0.38 μM had higher cell integrity than the H_2_O_2_-treated group (Figure 3 C). Similarly, compared to the H_2_O_2_-treated group, PRO-7a at doses ranging from 6.25 to 0.38 μM increased mitochondrial metabolism (Figure 3 D). PRO-7a had a neuroprotective action against H_2_O_2_-induced stress in PC12 cells at these doses; however, the concentration of 1.56 μM had the highest statistical significance; therefore, it was used in the next experiments. PC12 cells exposed to H_2_O_2_ at 0.5 mM for 20 h significantly decreased cell integrity to 74.58 ± 2.16 % and mitochondrial metabolism to 54.45 ± 3.64 % after treatment, compared to the control (Figure 3 B).

### Mitochondrial metabolism

**Figure 4.**
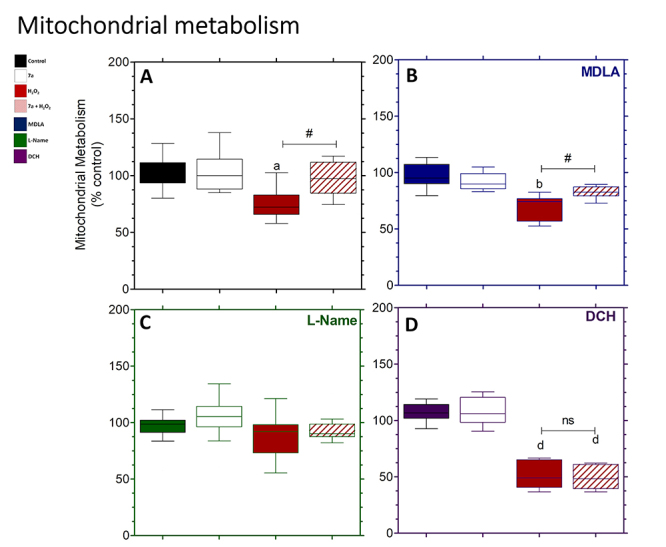
PRO-7a-mediated cytoprotection on the mitochondrial metabolism in neuronal PC12 cell. **(A)** PRO-7a attenuated the oxidative stress-induced changes in mitochondrial metabolism, using the MTT reduction assay. In PRO-7a-mediated cytoprotection, MDLA **(B)**, L-Name **(C)**, and DCH **(D)** were used to inhibit AsS and NOS, as well as block M1 mAChR, respectively. Data were shown box-and-whisker plots in % to control from three independent experiments in sextuplicate and analyzed by one-way ANOVA followed by Dunnett’s post-test. p < 0.05 for differences in relation to the control (a), MDLA (b), L-Name (c), and DCH (d) groups; p < 0.05 for differences between groups (#); not significant (ns). MTT: 3-(4,5-dimethylthiazol-2-yl)-2,5-diphenyltetrazolium bromide; MDLA: Methyl-DL-aspartic acid; L-Name: L-N^Ω^-nitroarginine methyl ester; DCH: Dicyclomine hydrochloride; AsS: Argininosuccinate synthase; NOS: Nitric oxide synthase; M1 mAChR: M1 muscarinic acetylcholine receptor.

Cells subjected to oxidative stress (H_2_O_2_ group) altered mitochondrial metabolism compared to controls, but this was restored when the cells were also treated with PRO-7a (7a + H_2_O_2_ group) (Figure 4 A). Cells treated only with MDLA or MDLA + 7a did not change mitochondrial metabolism, but when treated with MDLA + H_2_O_2_, on the other hand, mitochondrial activity was reduced. The MDLA + 7a + H_2_O_2_ group alleviated the reduction of H_2_O_2_-induced mitochondrial metabolism (Figure 4 B). There were no changes in mitochondrial metabolism in cells treated with only L-name in all groups studied (Figure 4 C). Cells treated with DCH + H_2_O_2_ reduced mitochondrial metabolism, but no effects were seen with DCH or DCH + 7a. Metabolism was lowered in cells treated with DCH + H_2_O_2_ or DCH + 7a + H_2_O_2_ compared to the control, but no significant difference was seen between these two groups (Figure 4 D). 

### ROS generation

**Figure 5.**
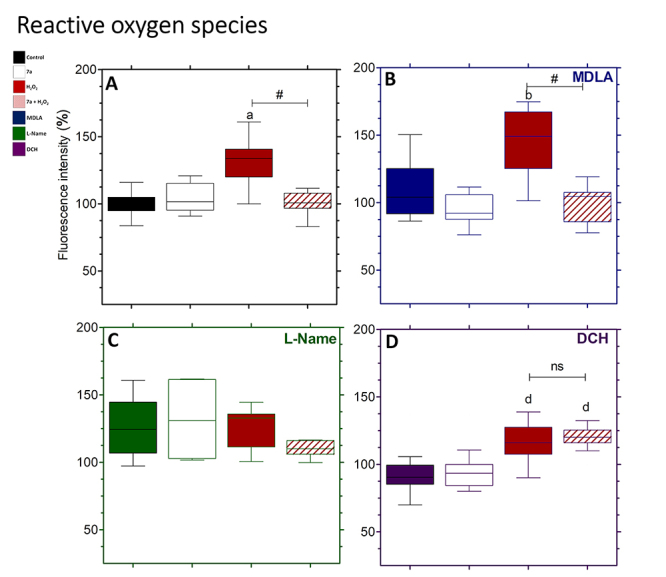
Reactive oxygen species (ROS) production during 7a-mediated cytoprotection in neuronal PC12 cells. **(A)** Cells treated with PRO-7a prevented ROS generation induced by oxidative stress, using H_2_DCF-DA assay. In PRO-7a-mediated cytoprotection, MDLA **(B)**, L-Name **(C)**, and DCH **(D)** were used to inhibit AsS and NOS, as well as block M1 mAChR, respectively. Data were shown box-and-whisker plots in % to control from three independent experiments in sextuplicate and analyzed by one-way ANOVA followed by Dunnett’s post-test. *p* < 0.05 for differences in relation to the control (a), MDLA (b), L-Name (c), and DCH (d) groups; p < 0.05 for differences between groups (#); not significant (ns). H_2_DCF-DA: 2’,7’ - dichlorodihydrofluorescein diacetate; MDLA: Methyl-DL-aspartic acid; L-Name: L-N^Ω^-nitroarginine methyl ester; DCH: Dicyclomine hydrochloride; AsS: Argininosuccinate synthase; NOS: nitric oxide synthase; M1 mAChR: M1 muscarinic acetylcholine receptor.

ROS levels were considerably greater in the H_2_O_2_ group than in the control group, although they were reduced by the 7a + H_2_O_2_ treatment (Figure 5 A). The PRO-7a fluorescence intensity was comparable to the control group (Figure 5 A). ROS levels were greater in cells treated with MDLA + H_2_O_2_ than in cells treated with MDLA alone, but lower in cells treated with MDLA + 7a + H_2_O_2_ (Figure 5 B). In all groups, the L-Name use did not raise the ROS levels (Figure 5 C). When compared to the H_2_O_2_ group (Figure 5 A), L-Name + H_2_O_2_ substantially reduced ROS levels (Figure 5 C). Cells treated with DCH + H_2_O_2_ or DCH + 7a + H_2_O_2_ produced more ROS than cells treated with DCH or DCH + 7a (Figure 5 D).

### Arginase indirect activity

**Figure 6.**
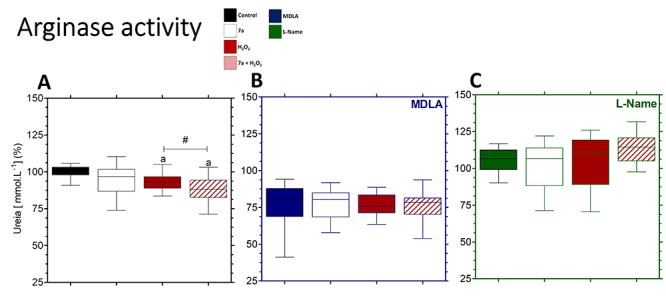
Arginase indirect activity in the PRO-7a-mediated cytoprotection in neuronal PC12 cells. **(A)** The PRO-7a restored the urea concentration levels in the presence of oxidative stress in the culture medium. **(B and C)** The AsS and NOS activities were inhibited by specific inhibitors, MDLA or L-Name, respectively. Data were shown box-and-whisker plots in % to control from three independent experiments in sextuplicate and analyzed by one-way ANOVA followed by Dunnett’s post-test. p < 0.05 for differences in relation to control (a), MDLA (b), and L-Name (c); p < 0.05 for differences between groups (#). AsS: argininosuccinate synthase; NOS: nitric oxide synthases; MDLA: methyl-DL-aspartic acid; L-Name: L-N^Ω^-nitroarginine methyl ester.

The oxidative stress caused by H_2_O_2_ treatment reduced urea levels in comparison to the control (Figure 6 A). Surprisingly, the 7a + H_2_O_2_ treatment decreased urea compared to the H_2_O_2_ group (Figure 6 A). MDLA treatment decreased urea levels in all groups (Figure 6 B). There was no significant difference between the MDLA + H_2_O_2_ and MDLA + 7a + H_2_O_2_ groups (Figure 6 B). L-Name increased urea concentration in all groups (Figure 6 C) compared to the control (Figure 6 A). Urea levels were not different in cells treated with L-Name + H_2_O_2_ or L-Name + 7a + H_2_O_2_ (Figure 6 C).

## Discussion

We found that PRO-7a, a bioactive heptapeptide described from *B. jararaca* crude venom analysis, demonstrated cytoprotection in neuronal PC12 cells with dopaminergic characteristics, but not in astroglial C6 cells in an H_2_O_2_-induced oxidative stress model *in vitro* for the study of neurodegenerative diseases. The PRO-7a-induced neuroprotection is mediated through M1 mAChR activation, which reduces oxidative stress indicators and neuron injury. Furthermore, unlike PRO-10c-mediated neuroprotection [10], PRO-7a did not alter AsS activity or L-arginine bioavailability to generate neuroprotective metabolites such as agmatine and polyamines that minimize oxidative stress-induced changes.

The H_2_O_2_-induced oxidative stress has been employed as a neurodegenerative model *in vitro,* which causes mitochondrial dysfunction, lipid peroxidation, cell membrane changes, and dead cells [42, 43]. In C6 and PC12 cells, H_2_O_2_ decreased the viability of cells in a concentration-dependent manner [44- 47], and it has been used to investigate the cytoprotection mediated by venom compounds of different species against H_2_O_2_-induced oxidative stress [14, 44, 47]. For the first time, we demonstrated that the PRO-7a-mediated cytoprotection in PC12 cells led to a significant reduction in oxidative stress damage in a dose-dependent manner. Despite this, the cytoprotection effects were not observed in the astroglial C6 cell line but enhanced H_2_O_2_-induced toxicity at concentrations lower than 0.38 μM, similar to what was demonstrated by the peptide fraction from *B. jararaca* snake venom, which contains a variety of PROs and also potentiated the H_2_O_2_-induced toxicity [14]. Astrocytes can act as one of the main sources of harmful ROS [48], generating new radicals that damage major cellular components under certain pathological conditions [49]. A C6 cell line is widely used as an astrocyte-like cell line to study astrocytic function [46], which responds quickly to external stimuli, such as H_2_O_2_, producing oxidative-nitrosative stress [46, 50]. The C6 cell sensibility to stimuli promoted by PRO-7a could explain the potentiation of oxidative stress-induced toxicity. However, in our cell viability investigation, PRO-7a did not show cytotoxic effects on C6 cells or neuronal cells (PC12 cells) at the conditions tested, and further experiments will be required to examine these effects.

**Figure 7.**
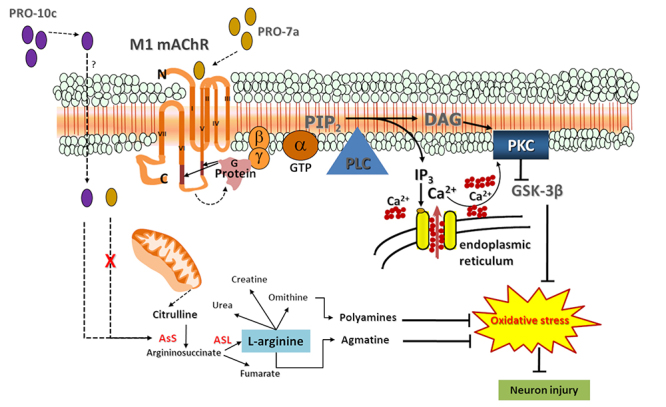
PROs-mediated neuroprotection mechanisms proposals. PRO-10c is internalized by an unknown mechanism, increasing L-arginine synthesis by raising AsS expression and activity. As a result of the elevated L-arginine content and metabolism, polyamines and agmatine are produced, which are neuroprotective chemicals in the oxidative stress response in neurodegenerative diseases [9, 10]. PRO-7a activates M1 mAChR, inducing a multitude of effects via M1 mAChR-mediated PKC and mitogen-activated protein kinase signaling, independent of increased L-arginine by AsS activation, triggering a cellular response to oxidative stress. AsS: Argininosuccinate synthetase; AsL: Argininosuccinate lyase; GTP: Guanosine triphosphate; M1 mAChR: M1 muscarinic acetylcholine receptor; PIP_2_: Phosphatidylinositol 4,5-bisphosphate; PLC: Phospholipase C; IP3: Inositol triphosphate; DAG: Diacylglycerol; PKC: Protein kinase C; GSK3β: Glycogen synthase kinase 3β.

The PROs-mediated neuroprotection with different structural and functional properties was studied on oxidative stress-conditioned damage in the human neuroblastoma SH-SY5Y cell line [9, 10, 51]. Interestingly, despite the similarity between the amino acid sequences of PROs, the distinct effects of oxidative stress markers in H_2_O_2_-induced toxicity have suggested that they can affect their targets via a variety of mechanisms [9, 10, 51]. PROs 7a and 10c were reported as potent neuroprotective peptides, improving cell viability and decreasing ROS generation, lipid peroxidation, and total glutathione in response to H_2_O_2_ damage in SH-SY5Y cells [9]. The neuroprotective effects of PRO-10c, an AsS activator [21], have been attributed to increased AsS expression and activity, improving L-arginine synthesis, and that its metabolism would lead to L-arginine metabolism products such as agmatine and polyamines, which have been widely shown to have neuroprotective action (Figure 7) in SH-SY5Y cells [10]. Querobino and collaborators also raise the hypothesis that AsS expression is also not implicated in the neuroprotective mechanism for PRO-7a peptides in SH-SY5Y cells [9], in contrast to PRO-10c [10]. For these reasons, the current work was also structured at the cytoprotective effects of PRO-7a in dopaminergic neuronal PC12 cells, as well as the role of L-arginine metabolite production via AsS activity regulation and the M1 mAChR receptor in PRO-7a-mediated neuroprotection.

The PRO-7a-mediated cytoprotection mechanisms were investigated using the concentration of 1.56 µM since it displayed the highest statistical significance compared to the other concentrations evaluated. The PRO-7a decreased mitochondrial metabolism, ROS generation, and arginase activity against oxidative stress-induced changes in PC12 cells. Based on the neuroprotective properties of L-arginine metabolites [26, 27, 30], we investigated their involvement in PRO-7a-mediated cytoprotection pathways using specific inhibitors, MDLA [10, 31] or L-Name [38] for two of the key enzymes in the L-arginine metabolic pathway, AsS and NOS, respectively. NOS enzyme catalyzes the synthesis of NO from L-arginine via a Ca^2+^/calmodulin-dependent mechanism [52, 53]. Because of its capacity to bind to the mitochondrial respiratory chain's cytochrome C oxidase enzyme with greater affinity and speed than oxygen, NO can be a main mitochondrial modulator in stressful settings, preventing apoptosis and regulating ROS generation [54- 56]. NO also could inhibit the interaction between a peroxide and a metal ion, reducing the generation of ROS and lowering lipid peroxidation [54]. When ROS and arginase activities were examined in our study, MDLA and L-Name did not block the cytoprotective effects of PRO-7a in PC12 cells, demonstrating that the mechanism involved in oxidative stress protection is independent of AsS activity, L-arginine bioavailability, and the generation of its neuroprotective metabolites.

Studies have revealed that PRO-7a is an M1 mAChR agonist, which is of critical relevance given the extensive research that focused on finding such a ligand [24, 57, 58]. The peptide specifically activates [Ca^2+^]_I_ transients in CHO and neuronal P19 cells expressing the M1 mAChR subtype, which are inhibited in cells pretreated with the M1 mAChR specific antagonist pirenzepine [24]. The PRO-7a also improved anxiolytic and antidepressant-like actions in rats, as well as increased mobility and exploration, and these effects appear to be partially dependent on M1 mAChR activation [57, 58]. Then, since PC12 cells express the M1 mAChR endogenously [59, 60], we investigated the potential involvement of cholinergic receptors in PRO-7a-mediated neuroprotection via activation of M1 mAChR, using the competitive antagonist DCH [39] to suppress receptor activity in our neuroprotective assay with PRO-7a in PC12 cells. 

The neuroprotective effects in the brain by cholinergic activation are mostly attributed to M1 mAChR subtype activation [61], and it has emerged as a pivotal therapeutic target for neurodegenerative diseases [34]. A great number of cholinergic muscarinic agonists have been evaluated clinically in the last few years [34, 62, 63]. Furthermore, the inherent selectivity achieved by targeting allosteric binding sites (distinct from the acetylcholine binding site) has inspired the pharmacological approach to targeting the M1 receptor, leading to the development of both positive allosteric modulators (PAMs) and direct-acting allosteric agonists [34]. In our investigation, PRO-7a-mediated neuroprotection in PC12 cells did not prevent oxidative stress-induced neurotoxicity when the DCH antagonist of M1 mAChRs was employed. It indicates that the neuroprotective effects of PRO-7a against H_2_O_2_-induced oxidative stress occur through M1 mAChR activation, independent of AsS activity and L-arginine bioavailability (Figure 7). Then, we proposed that, unlike PRO-10c, specific M1 mAChR via G-protein/PLC/PKC signalizing might be involved in the neuroprotective effects of PRO-7a, which inhibit GSK3β, decreasing oxidative stress and neuron injury.

## CONCLUSIONS

Taken together, PRO-7a from *B. jararaca* snake venom displayed cytoprotective effects against H_2_O_2_-induced oxidative stress in neuronal PC12 cells but not in astroglial C6 cells. In PC12 cells, PRO-7a-mediated neuroprotection was characterized by the decrease in oxidative stress markers and was dependent on M1 mAChR-mediated G-protein/PLC/PKC signaling, thereby alleviating H_2_O_2_-induced PC12 cell injury. In addition, blocking two key enzymes in the L-arginine metabolic pathway, AsS, and NOS, did not inhibit PRO-7a-mediated neuroprotection, implying that its mechanism is independent of the production pathway of L-arginine metabolites, which is neuroprotective in contrast to what was reported by PRO-10c. These findings provide a snake venom peptide M1 muscarinic receptor agonist that could be used for basic research and neuropharmacological applications.

## Abbreviations

ACE: angiotensin-converting enzyme; ANOVA: one-way analysis of variance; AsL: argininosuccinate lyase; AsS: argininosuccinate synthetase; BPPs: bradykinin potentiating peptides; C6: astrocyte-like cell line; Ca^2+^: Calcium; CHO: chinese hamster ovary cell line; CNS: central nervous system; DAG: Diacylglycerol; DCH: dicyclomine hydrochloride; DMEM: Dulbecco’s modified Eagle’s medium; DMSO: dimethyl sulfoxide; <E: pyroglutamic residue; EDTA: Ethylenediaminetetraacetic acid; FBS: fetal bovine serum; GSK3β: glycogen synthase kinase 3β; GTP: Guanosine triphosphate; H_2_DCF-DA: 2’,7’ - dichlorodihydrofluorescein diacetate; H_2_O_2_: Hydrogen peroxide; Ham-F-10: Nutrient Mixture F-10 Ham; HPLC: high performance liquid chromatography; L-Name: L-NΩ-Nitroarginine methyl ester; M1 mAChR: M1 muscarinic acetylcholine receptor; MALDI-TOF MS: matrix assisted laser desorption/ionization time-of-flight mass spectrometry; MAPK: mitogen-activated protein kinase; MDLA: α-Methyl-DL-aspartic acid; MTT: 3-(4,5-dimethylthiazol-2-yl)-2,5-diphenyltetrazolium bromide; NO_2_: Nitrite; NO: nitric oxide; NOS: nitric oxide synthase; P19 - embryonal carcinoma cells; P: proline; PAMs: positive allosteric modulators; PBS - phosphate buffered saline; PC12: neuronal cell line derived from a transplantable rat pheochromocytoma; PIP2: Phosphatidylinositol 4,5-bisphosphate; PKC: protein kinase C; PLC: phospholipase C; PROs: bradykinin-potentiating peptides; ROS: reactive oxygen species; SD: standard deviation; SH-SY5Y: human neuroblastoma cell line; SOD: superoxide dismutase.
